# Constrained Fitness Landscape Analysis of Capacitated Vehicle Routing Problems

**DOI:** 10.3390/e24010053

**Published:** 2021-12-28

**Authors:** Sebastián Muñoz-Herrera, Karol Suchan

**Affiliations:** 1 Departamento de Ingeniería Industrial, Universidad Católica de la Santísima Concepción, Alonso de Ribera 2850, Concepcion 4090541, Chile; 2 Facultad de Ingeniería y Ciencias, Universidad Adolfo Ibáñez, Av. Diagonal las Torres 2640, Santiago 7941169, Chile; 3 Facultad de Ingeniería y Ciencias, Universidad Diego Portales, Av. Ejército Libertador 441, Santiago 8370191, Chile; karol.suchan@mail.udp.cl; 4 Faculty of Applied Mathematics, AGH University of Science and Technology, al. A. Mickiewicza 30, 30-059 Krakow, Poland

**Keywords:** vehicle routing problem, Fitness Landscape Analysis, information analysis, statistical analysis, feasibility analysis, Principal Component Analysis, Multinomial Logistic Regression

## Abstract

Vehicle Routing Problems (VRP) comprise many variants obtained by adding to the original problem constraints representing diverse system characteristics. Different variants are widely studied in the literature; however, the impact that these constraints have on the structure of the search space associated with the problem is unknown, and so is their influence on the performance of search algorithms used to solve it. This article explores how assignation constraints (such as a limited vehicle capacity) impact VRP by disturbing the network structure defined by the solution space and the local operators in use. This research focuses on Fitness Landscape Analysis for the multiple Traveling Salesman Problem (m-TSP) and Capacitated VRP (CVRP). We propose a new Fitness Landscape Analysis measure that provides valuable information to characterize the fitness landscape’s structure under specific scenarios and obtain several relationships between the fitness landscape’s structure and the algorithmic performance.

## 1. Introduction

The Capacitated Vehicle Routing Problem (CVRP) (the reader is referred to the monograph by Toth and Vigo [1] for an introduction to Vehicle Routing Problems) is a classical combinatorial optimization problem that has attracted much attention given its many applications and the difficulty of obtaining good solutions. (Even TSP, the simplest variant of VRP, is NP-complete—for more detailed complexity considerations the reader is referred to [1]). Nowadays, the wide range of variants originating from the problem is interesting both for academia and industry.

CVRP models the following situation: Given a central depot, a set of customers with known demands, and a set of vehicles of equal capacity, we need to choose a set of economic routes that allows delivering the amount of product requested by the customers without exceeding the vehicle capacity, where each customer is visited exactly once. This situation is quite distant from real-life distribution systems, but provides a context for different formulations and variants which has led to the development of a wide range of algorithms that yield good solutions.

Historically, Vehicle Routing Problems (VRP) solution methods have constantly evolved due to computational advances, ranging from the analysis of exact algorithms to sophisticated metaheuristics. Vidal et al. [2] provides a detailed review of the evolution of VRP solution methods for variants that include many additional characteristics and decisions, called *attributes*. In this context, hybrid algorithms have exhibited the best performance for VRP, both from the solution quality and the computational time points of view. These algorithms often use local search and population-based methods.

The design of algorithms for combinatorial optimization problems and, in particular, for VRP has suffered from the so-called *Competitive Paradigm* [3] in which researchers develop new methods guided by the computational results, often without questioning the algorithm’s operation or the structure of the given problem or instance. Faced with this deficiency, arises the so-called Fitness Landscape Analysis (FLA), which has as the main objectives to understand the algorithm’s performance, to give a topological description of the search space, and to give an idea about the differences and similarities among problems and instances [4].

The following article aims to give a baseline for the study of different variants of VRP with FLA techniques, by analyzing trajectories generated by random walks in search spaces obtained using different representations and local operators on constrained and unconstrained problems. The foundations of VRP are associated with the extensive research on Traveling Salesman Problem (TSP) and its multiple salesmen version (m-TSP). It seems interesting to study the transitions between different variants of VRP starting from m-TSP.

The contributions of the article are twofold. Firstly, it studies the effect of constraints in fitness landscapes based on VRP. In this context, we present a unified set of FLA measures that incorporates statistical, information and feasibility techniques to characterize the landscape. Additionally, it presents a new measure based on the information content curve’s shape that helps to achieve high precision in the task of classifying the difficulty of instances for solving VRP by local search algorithms.

The structure of the article is the following: Section 2 presents a brief description of the main VRP variants studied in this work and some local operators often used in solution algorithms for this type of problems. Section 3 presents a short review of the literature associated with FLA and its scarce applications to VRP. It also introduces a new FLA measure. Section 4 describes the experimental design: the instances selected, the fitness landscapes created and the analysis techniques applied. Further, Section 5 presents the data obtained from sampling the fitness landscapes, together with the results of statistical and machine learning analyses performed on them. Finally, in the Section 6 we briefly discuss the contributions of this work.

## 2. From m-TSP to Multi-Attribute VRP

It is common to find in the literature that TSP is a particular case of VRP in which the number of vehicles is one and the capacity is infinite [1]. The other way around, from the search space perspective, a multiple salesman version of TSP (m-TSP) can be seen as the origin for many VRP variants. Adding attributes to m-TSP limits the feasible solution space—it reduces the set of possible solutions that search algorithms (restricted to analyzing only feasible solutions) can visit. In this work, we focus on m-TSP and CVRP, which corresponds to a transition from an unconstrained base problem to problems restricted at various levels of vehicle capacity.

In this section, we present Mixed-Integer Linear Programming (MILP) formulations of m-TSP and CVRP. Among many alternatives studied in the literature, we choose formulations based on three indexes because they keep a direct relationship with the representations we use to define the search space for local search. For this research, we propose the use of two types of path representations: *Delimeters* (an array that contains one substring per trip and k+1 copies of the depot, for a problem with *k* vehicles, used as trip delimiters [5]) and the classical *Giant-Tour* representation [6]. Section 4.2 presents more details about the representations.

### 2.1. Multiple-Traveling Salesman Problem

Although TSP is one of the combinatorial optimization problems most studied in the literature, m-TSP has received less attention than it should. This problem can be seen as a variant of CVRP with the vehicle capacity equal to the total demand of customers, thus making it practically unrestricted. Therefore, it can be considered a relaxation of CVRP [7]. A MILP formulation representing m-TSP arises under the following definitions: Let G=(V,E) be a directed graph, where V={0,1,⋯,n} is the set of customers, including the unique depot represented as the vertex zero. (The reader is referred to the textbook by Diestel [8] for standard notions, notation and results in graph theory.) Also, let $c_{i,j}$ be the cost of traveling from *i* to *j* associated to the arc (i,j), for any pair of customers *i* and *j*. In many cases, this represents the distance or travel time. In the instances studied in this paper, the cost corresponds to the Euclidean distance between the customers’ locations on the plane, so the cost function *c* is symmetric. The binary decision variables are $x_{i,j,k}$, where 1 indicates that the arc (i,j) is used by the vehicle *k*, k∈K, where *K* is the set of available vehicles. Thus, there are |K| vehicles available to visit exactly once each of the customers. The vehicle capacity is not considered. The formulation is as follows:

(1) $$\mathrm{Minimize}\;\sum_{k\in K}\sum_{i,j\in V}c_{i,j}x_{i,j,k}$$

Subject to:

(2) $$\begin{array}{cc} \sum_{i\in V}\sum_{k\in K}x_{i,j,k}=1 & \forall j\in V\backslash \{0\} \end{array}$$

(3) $$\begin{array}{cc} \sum_{i\in V}x_{i,h,k}=\sum_{j\in V}x_{h,j,k} & \forall h\in V\backslash \{0\},k\in K \end{array}$$

(4) $$\begin{array}{cc} \sum_{j\in V\backslash \{0\}}x_{0,j,k}=1 & \forall k\in K \end{array}$$

(5) $$\begin{array}{cc} \sum_{i,j\in S}x_{i,j,k}\leq \left|S\right|-1 & \forall k\in K,S\subseteq V\backslash \{0\}:|S|\geq 2 \end{array}$$

(6) $$\begin{array}{cc} x_{i,j,k}\in \left\{0,1\right\} & \forall i,j\in V,k\in K \end{array}$$

The objective function (1) minimizes the total cost associated with the arcs chosen in the solution. The expressions (2) indicate that each customer must be visited exactly once by a vehicle. In constraints (3), a balance of the incoming and outgoing flow is assured for each customer. The constraints set (4) indicates that each vehicle must leave the depot exactly once. Furthermore, in (5), sub-tour-elimination constraints apply. Finally, the constraints (6) ensure the nature of the decision variables. It is important to note that there are many m-TSP formulations studied in the literature. In this brief presentation, we consider only the one that can be used with greater flexibility in relation to the MILP formulation of CVRP used in this paper.

### 2.2. Capacitated Vehicle Routing Problem

The most studied VRP variant is CVRP, briefly presented in the introduction. Although CVRP’s use is mainly academic, it constitutes the basis for formulating different extensions that seek to emulate real-life dispatch systems. A formulation for CVRP adds the following constraints to the m-TSP [9]:

(7) $$\sum_{i\in V\backslash \{0\}}q_{i}\sum_{j\in V}x_{ijk}\leq Q,\;\forall k\in K\;$$

The set of expressions (7) represents the constraints associated with the vehicle capacity *Q* and the customers’ demand $q_{i}$ associated with each customer *i*, i∈V\{0}. Each vehicle cannot exceed its capacity.

### 2.3. Local Operators in Vehicle Routing Problems

The design of algorithms for VRP often considers local search. In this context, a specific local operator defines the *neighborhood* N(x) associated with a solution *x*. Thus, the function *N* maps any solution x∈X to the set N(x) of solutions that can be obtained from *x* by a single application of the local operator. If y∈N(x), then *y* is a *neighbor* of the solution *x*.

**Figure 1:**
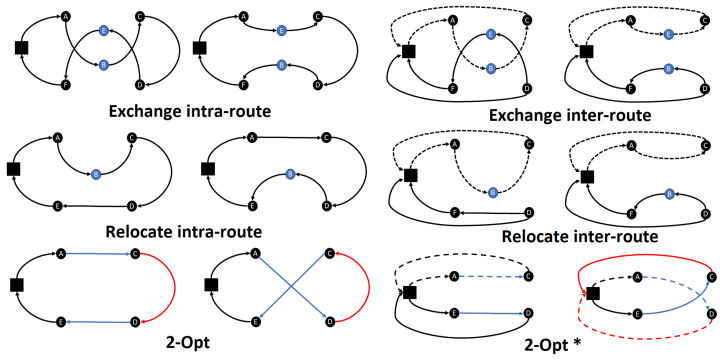
Local operators for vehicle routing problems.

For VRP, the local operators most frequently used are strongly influenced by local operators created for TSP. The most common ones are Exchange, Relocate and 2-Opt , and their adaptations to sequencing several routes simultaneously (Figure 1). For details, see [10] for a review of route construction and local search algorithms.

- Relocate operator for VRP is a generalization of the insertion move for TSP: a customer is removed from his current position and put in another one. This move is executed within the same route (intra-route) or between two routes (inter-route).
- The Exchange operator for VRP swaps the positions of two customers, keeping the others constant. This operator is applied both inter and intra-route.
- 2-Opt is the local operator most used in TSP-related heuristics. It inverts customer segments and reconnects them. An application of this operator is equivalent to removing two arcs and creating two new ones. It is generalized in VRP as the 2-Opt* operator. The basic idea is to combine two routes so that the latest customers of one route are introduced after the first customers of the other one, maintaining the routes’ orientations.

Sometimes also hybrid operators are considered, for example, Relocate or Exchange or 2-Opt. In such cases, the neighborhood is the union of the neighborhoods of the individual operators. These operators are the basic building blocks of many powerful metaheuristics for VRP [11].

The choice of a local operator implies a *fitness landscape*: a triplet (X,N,f), where *X* is the solution space, *f* is the fitness function and *N* is the neighborhood function defined by the operator [12]. We focus on neighborhoods that are symmetric, i.e., $x_{j}\in N\left(x_{i}\right)$ if and only if $x_{i}\in N\left(x_{j}\right)$, since they are common in VRP. Sometimes we will use the term *search space* to refer just to the structure of the graph defined by (X,N): G=(X,E), where $\{x_{i},x_{j}\}\in E$ if $x_{i},x_{j}\in X$ and $x_{j}\in N\left(x_{i}\right)$.

## 3. Background on Fitness Landscape Analysis

Research on FLA is generally related to simulation-based methods, which collect data during the search process to infer the nature of a fitness landscape. In this context, most studies have been based on statistical (e.g., [13]) and information (e.g., [14]) FLA measures. Additionally, Malan et al. [15] propose complementary ways of characterizing a fitness landscape’s structure using FLA measures related to feasibility.

For the presentation of FLA measures, let us focus on maximization problems. Naturally, a minimization problem can be converted easily to a maximization problem by changing the sign of the cost function.

### 3.1. Statistical Analysis

Often, the size of the solution space grows exponentially in terms of the instance size, and so the exhaustive searching is not a practical option. For this reason, choosing a sampling method is the only feasible way to explore the fitness landscape. The most common choice is to rely on random walks, since they give a perspective on the context in which local-search-based algorithms perform their exploration – but in a somehow “algorithm-agnostic” way.

Historically, the first approach towards understanding a fitness landscape’s structure was through measurements of the degree of correlation between fitness function values at nearby points. When considering a sequence ${\left\{x_{t}\right\}}_{t=1}^{n}=\left\{x_{1},x_{2},\ldots ,x_{n}\right\}$ of solutions (usually obtained from a random walk in the search space), its respective sequence of fitness values ${\left\{f\left(x_{t}\right)\right\}}_{t=1}^{n}=\left(f\left(x_{1}\right),f\left(x_{2}\right),\ldots ,f\left(x_{n}\right)\right)$ is defined. If the sequence values $f_{t}$ are considered random variables, the potential for using statistical tools from time series analysis becomes evident. In this context, Weinberger [13] points out that, if the sequence describes a stationary stochastic process in which the statistics do not change over time, the Autocorrelation Function ρ(s) is defined as:

(8) $$\rho \left(s\right)=\rho \left(f_{t},f_{t+s}\right)=\frac{E\left[f_{t+s}f_{t}\right]-E\left[f_{t}\right]E\left[f_{t+s}\right]}{var[f_{t}]}$$

The statistical FLA measures allow us to know if a fitness landscape is smooth or rugged, depending on the autocorrelation of the sequences. A fitness landscape is smooth if the autocorrelation is high and rugged if the autocorrelation is low. The value of ρ(1) is especially relevant in ruggedness analysis since it provides a notion of the relationship between a solution and its direct neighborhood. For many problems, empirical autocorrelation functions of random walks show an exponential decay in the form of $\rho \left(s\right)=e^{-s/\tau }$. Thus the Correlation Length (CL) $\tau =-\frac{1}{ln(\rho (1))}$, where τ indicates the distance (number of steps) in which solutions begin to be uncorrelated. Analogously, ref. [16] present the Autocorrelation Coefficient (AC) given by $\xi =\frac{1}{1-\rho (1)}$.

### 3.2. Information Analysis

Information analysis introduced by Vassilev [14] proposes a modification to the statistical FLA measures. The sequence $f={\left\{f_{t}\right\}}_{t=1}^{n}=\left(f\left(x_{1}\right),f\left(x_{2}\right),\ldots ,f\left(x_{n}\right)\right)$ is encoded into a new one based on trend changes of the fitness function with a degree of tolerance, named ε. The new sequence of signs $s\left(\varepsilon \right)=\left(s_{1},s_{2},\ldots ,s_{n-1}\right)$ is constructed as:

(9) $$s_{i}\left(\varepsilon \right)=\Psi_{f_{t}}\left(i,\varepsilon \right)=\left\{\begin{array}{cc} \bar{1} & \;\;\mathrm{if}\;f_{i+1}-f_{i}\leq -\varepsilon \\ 0 & \;\;\mathrm{if}\;|f_{i+1}-f_{i}|<\varepsilon \\ 1 & \;\;\mathrm{if}\;f_{i+1}-f_{i}\geq \varepsilon \end{array}\right.$$

Modifying the value of ε is used to study the fitness landscape’s behavior at different resolution levels.

**Figure 2:**
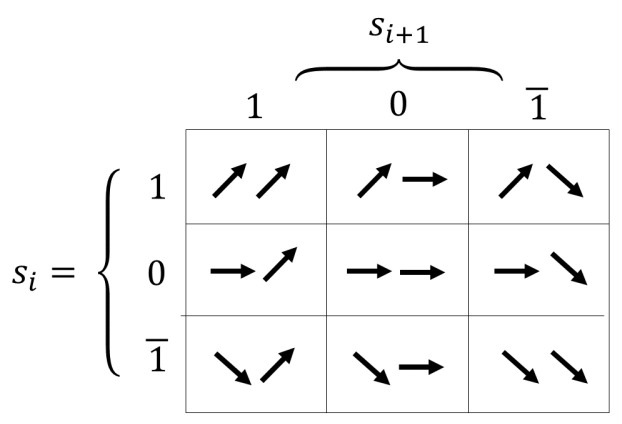
Types of observable objects.

From the information theory perspective, we consider the following definitions:

- An *object* is composed of two consecutive signs. Consider choosing at random two consecutive elements within a sequence, and the object as the corresponding random variable assigned to the subsequence. Figure 2 shows a representation of all objects that could be observed in a fitness landscape.
- *Self-information* is the information associated to a single object. An object of low probability has high self-information and provides a large amount of information.
- *Entropy* measures the average amount of self-information that all objects contribute to a system.

We use entropic FLA measures to describe the structure of the fitness landscape. In this context, we consider a fitness landscape defined as a vertex-weighted graph $G=(V,E_{\vartheta },f)$, where *V* and $E_{\vartheta }$ are the sets of vertices (solutions) and edges (transitions though a single use of the local operator), respectively, *f* is the fitness function and ϑ is the local operator that specifies the neighborhood $N_{\vartheta }$.

Consider the partition of $E_{\vartheta }$ into $E_{\vartheta }^{=}$ and $E_{\vartheta }^{!}$—the subsets of edges connecting vertices with equal and different fitness values, respectively. Let $G^{=}=\left(V,E_{\vartheta }^{=},f\right)$ and $G^{!}=\left(V,E_{\vartheta }^{!},f\right)$, correspondingly. Thus, the fitness landscape’s structure can be interpreted using a set of definitions based on the graph perspective:

- A vertex v∈V is a *global optimum* if v∈V|∀w∈V,f(v)≥f(w). Thus, a global optimum is independent of the operators.
- A vertex v∈V is a ϑ-*local optimum* if $v\in V|\forall w\in N_{\vartheta }\left(v\right),f\left(v\right)\geq f\left(w\right)$.
- A vertex v∈V is a ϑ-*peak* if $v\in V|\forall w\in N_{\vartheta }\left(v\right),f\left(v\right)>f\left(w\right)$.
- A subset P⊂V of two or more vertices is a ϑ-*plateau* if they form a connected component in $G^{=}$. (The proposed definition of a plateau involves a connected set of at least two vertices with the same fitness. This definition is different from the common 3-D concept of a plateau, which considers an area where a step in any direction generally will not result in a change of fitness. This approach proposes an adaptation to analyze the plateaus using a sequence of fitness values obtained from a walk in the search space generated by the local operator ϑ).
- The ϑ-*basin of attraction* $B_{\vartheta }\left(v\right)$ of a vertex *v* is the set of vertices from which *v* is reachable using a path of strictly increasing fitness. More formally, $B_{\vartheta }\left(v\right)=\left\{w\in V\right|\exists v_{0},v_{1},\ldots ,v_{n-1},v_{n}$, with $v_{i+1}\in N_{\vartheta }\left(v_{i}\right)$ and $f\left(v_{i+1}\right)>f\left(v_{i}\right)$ for all *i*, 0≤i≤n−1, and $v_{0}=w,v_{n}=v\}$.

When it does not lead to confusion, we will use the terms *peak*, *local optimum*, *plateau* and *basin of attraction*, without specifying the local operator.

In information analysis, these general concepts defined on the entire search space are extended to only what can be observed in the sequence of solutions under study. This analysis describes the notions of rugged and smooth areas based on the objects. In particular, 01, 10, $0\bar{1}$ and $\bar{1}0$ are considered *rugged objects with a neutral step*, $1\bar{1}$ and $\bar{1}1$ are *rugged objects without neutral steps*, 11 and $\bar{1}\bar{1}$ are *smooth objects without neutral steps* and 00 is a *smooth object with neutral steps*. The main objective of information analysis is to get a hint of the number of local optima, their distribution in the search space and their magnitudes, based on information FLA measures. In this context, Vassilev [14] adds two additional concepts related to modality:

- A peak with a small basin of attraction is called an *isolated optimum*. The size of its basin of attraction specifies the *degree of isolation* of a particular optimum. The smaller the basin of attraction, the higher the degree of isolation.
- A set of peaks is a *group of optima* if they belong to the same connected component of $G^{!}$.

#### 3.2.1. Information Stability

Information FLA measures depend on the parameter ε. When the parameter is zero, all the changes in the value of the fitness function are distinguished. Conversely, if the ε is too large, the entire sequence obtained is a neutral walk (all signs are equal to 0). One way to describe the fitness landscape’s structure is to use the maximum value of resolution ε for which the random walk is not completely neutral yet. This value equals the maximum difference of fitness function value between two neighbors within the random walk. As such, Information Stability $\varepsilon^{*}$ is calculated as $max_{i=1}^{n-1}\left|f\left(x_{i+1}\right)-f\left(x_{i}\right)\right|$.

#### 3.2.2. Partial Information Content

Vassilev [14] considers that the fitness landscape’s modality is related to (1) the number of local optima, (2) the number of groups of optima and (3) the size of the basins of attraction. Horn & Golober [17] argue that a large number of local optima by itself does not necessarily imply difficulty for algorithms. For this reason, the concept of modality used by Vassilev includes additional characteristics associated with groups of optima and basins of attraction.

In order to investigate (1) the number of local optima, we use a new sequence $s^{*}\left(\varepsilon \right)$ obtained from s(ε) by eliminating the *non-essential parts* in s(ε): values that are equal 0 or are repeated at consecutive positions. The result is $s^{*}\left(\varepsilon \right)=\left(s_{i_{1}},s_{i_{2}},\ldots ,s_{i_{\mu }}\right)$, a sequence of length μ, where $s_{i_{j}}\neq 0$ and $s_{i_{j}}\neq s_{i_{j+1}}$, for all *j*, 1≤j≤μ−1. Thus, μ (notice that μ≤n) represents the fitness landscape’s ruggedness and serves to compute Partial Information Content M(ε) as the length of $s^{*}\left(\varepsilon \right)$ scaled to the interval [0,1].

(10) $$M\left(\varepsilon \right)=\frac{\mu }{n}$$

In this way, M(ε)=0 if the length of the new sequence is 0, which indicates that the entire original sequence has a smooth structure (with or without neutral steps) (To be precise, if the entire original sequence has a smooth structure without neutral steps, then M(ε) only tends to 0 as the length of the original sequence tends to infinity, as the length of the reduced sequence is 1). On the opposite, M(ε) is 1 when the entire sequence is composed of peaks.

#### 3.2.3. Information Content and Density-Basin Information

Information Content and Density-Basin Information are entropic FLA measures that describe the frequency and diversity of objects formed by consecutive values in the sequence s(ε). For this, we compute the relative frequency $P_{\left[pq\right]}=\frac{n_{\left[pq\right]}}{n_{Total}}$, where $n_{\left[pq\right]}$ is the number of occurrences of the object pq in the sequence and $n_{Total}$ is the total length of the sequence, for each pair of signs *p* and *q* that may appear consecutively in s(ε).

In the case of Information Content, we consider only pairs of signs that correspond to a change in tendency (rugged objects). Remember that $p,q\in \{\bar{1},0,1\}$ as shown in Figure 2. Thus, Information Content can be calculated as:

(11) $$H\left(\varepsilon \right)=-\sum_{p\neq q}P_{\left[pq\right]}log_{6}P_{\left[pq\right]}$$

The use of base six logarithms yields a measure on the scale [0,1]—since the number of different object types that meet the condition is precisely 6. Note that, for a specific value of ε, H(ε) describes the frequency and the diversity of rugged objects.

Recall the definition of signs (Equation (9)). With an increase in the value of ε from $\varepsilon^{\prime }$ to $\varepsilon^{\prime \prime }$, the number of rugged objects may increase. A smooth object without neutral steps (i.e., 11 or $\bar{1}\bar{1}$) may become a rugged object with a neutral step. For example, $s_{i}^{\prime }s_{i+1}^{\prime }=11$, where $\varepsilon^{\prime }<\varepsilon^{\prime \prime }\leq f_{i+1}-f_{i}$ and $\varepsilon^{\prime }\leq f_{i+2}-f_{i+1}<\varepsilon^{\prime \prime }$, becomes $s_{i}^{\prime \prime }s_{i+1}^{\prime \prime }=10$. The number of rugged objects may also decrease. A rugged object with a neutral step can become a smooth object with neutral steps (i.e., 00). For example, for $s_{j}^{\prime }s_{j+1}^{\prime }=10$, where $f_{j+2}-f_{j+1}<\varepsilon^{\prime }\leq f_{j+1}-f_{j}$ and $f_{j+1}-f_{j}<\varepsilon^{\prime \prime }$, becomes $s_{j}^{\prime \prime }s_{j+1}^{\prime \prime }=00$. In a similar way, a rugged object without neutral steps may become a smooth object with neutral steps. Finally, there are transformations that keep the number of rugged objects. A rugged object without neutral steps may become a rugged object with a neutral step; or a smooth object without neutral steps may become a smooth object with neutral steps. No other transitions are possible. In particular, the number of null signs in s(ε) never decreases with an increase in ε, and so a smooth object $s_{k}^{\prime }s_{k+1}^{\prime }$ with neutral steps in $s(\varepsilon^{\prime })$ remains the same in $s(\varepsilon^{\prime \prime })$ for all $\varepsilon^{\prime \prime }>\varepsilon^{\prime }$. The number of rugged objects without neutral steps and the number of smooth objects without neutral steps never increase.

In this context, Malan & Engelbrecht [18] propose the scalar measure of Maximum Information Content (MIC) ${\mathrm{Max}}_{\varepsilon \in [0,\varepsilon^{*}]}H\left(\varepsilon \right)$ for quantifying the fitness landscape’s ruggedness—a compact way of describing the curve defined by H(ε) as a function of ε. Notice that it is enough to consider values of ε lesser or equal Information Stability $\varepsilon^{*}$, since beyond this point, the sequence obtained is a neutral walk (Information Content equal zero).

For example, consider a trajectory *f* without neutral steps. So we have only one group of optima, no plateaus, some rugged objects without neutral steps, some smooth objects without neutral steps and no objects of other type. In this case, if we start from ε=0, with a gradual increase Δε>0, the frequencies of rugged objects with a neutral step may increase, but so the frequency of smooth objects with neutral steps. Thereby, when the MIC value is reached, the expected value of self-information of rugged objects is the highest. Therefore, in the interval [0,argmax(H(ε))], the frequency and variety of rugged objects increase due to an increase in the number of neutral steps. In this section, transformations into rugged objects with a neutral step predominate over transformations into smooth objects with neutral steps. On the contrary, after reaching MIC, the frequency and variety of rugged objects diminishes, so the curve begins to decrease from ε=argmax(H(ε)). Thus, in the decreasing section of H(ε), transformations into smooth objects with neutral steps predominate over transformations into rugged objects with a neutral step. Note that in fitness landscapes where smooth objects with neutral steps are more common than smooth objects without neutral steps, there is no increasing section in H(ε) and MIC is reached for ε=0.

Furthermore, it is possible to explore the smoothness of a fitness landscape from the perspective of basins of attraction, using *Density-Basins Information* h(ε)—a modification of H(ε) in which we only consider the objects that keep the tendency (smooth objects):

(12) $$h\left(\varepsilon \right)=-\sum_{p=q}P_{\left[pq\right]}log_{3}P_{\left[pq\right]}$$

**Figure 3:**
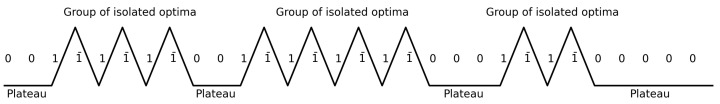
Sequence of signs s(ε) based in a trajectory $f_{2}$ composed only by groups of isolated optima.

For instance, consider a trajectory $f_{2}$ over a fitness landscape composed only of *groups of isolated optima*, i.e., $P_{\left[1,1\right]}=P_{\left[\bar{1},\bar{1}\right]}=P_{\left[1,0\right]}=P_{\left[0,\bar{1}\right]}=0$. Thus, each basin of attraction is the smallest possible, but there exist neutral steps that delimit groups of optima, as shown in Figure 3.

In this context, we denote the number of peaks as *m*, the number of groups of optima as *k* and the total length of the trajectory as *n*. In Figure 3, the probability of occurrence of respective objects are $P_{\left[1,\bar{1}\right]}=\frac{m}{n}$; $P_{\left[\bar{1},1\right]}=\frac{m-k}{n}$ and $P_{\left[0,1\right]}=P_{\left[\bar{1},0\right]}=\frac{k}{n}$; and $P_{\left[0,0\right]}=\frac{n-2m-k}{n}$. We obtain the following information FLA measures:

(13) $$\begin{array}{cc} M= & \frac{2m}{n} \end{array}$$

(14) $$\begin{array}{cc} H= & 2\frac{k}{n}log_{6}\left(\frac{n}{k}\right)+\frac{m-k}{n}log_{6}\left(\frac{n}{m-k}\right)+\frac{m}{n}log_{6}\left(\frac{n}{m}\right) \end{array}$$

(15) $$\begin{array}{cc} h= & \frac{n-2m-k}{n}log_{3}\left(\frac{n}{n-2m-k}\right) \end{array}$$

As shown in Expression (14), fitness landscapes with different numbers of local optima could have the same Information Content, depending on the number of groups. Furthermore, Expression (15) only considers the probability of occurrence of smooth objects with neutral steps. In this way, if the *h* value grows, then *k* decreases (for a fixed *m*). So the number of groups of optima decreases and the density of peaks increases. Thus, Density-basin information provides an idea about the number of peaks per group of optima.

In contrast, consider a similar fitness landscape, in which all the peaks are not isolated ($P_{\left[\bar{1},\bar{1}\right]},P_{\left[1,1\right]}>0$). Thus, Expression (15) will include the probability of smooth objects without neutral steps, increasing the variety of smooth objects observed in the fitness landscape. Consequently, a high value of h(ε) could indicate large basins of attraction or high density of peaks. In either case, h(ε) is capable of complementing other information-based FLA measures. However, note that h(ε) cannot give information about the characteristics of plateaus nor the size of basins of attraction, at least not directly.

### 3.3. Feasibility Analysis

An essential aspect of combinatorial optimization problems is to deal with constraints, and thus, with infeasible solutions. On the other hand, statistical and information analysis techniques use random walks restricted to visiting only feasible solutions, even if the definition of the constrained search space is strongly related to the unconstrained ground space. Therefore, they cannot explain the proportion of feasible solutions or the disjunction of feasible and infeasible zones within the ground search space. For feasibility analysis, we consider random walks that visit both feasible and infeasible solutions. In this context, Malan et al. [15] present two relevant FLA measures to characterize a constrained search space: Feasibility Ratio (FsR) and Ratio of Feasible Boundary Crossings (RFBC).

For the feasibility analysis in this research, the random walks are performed on the search space of the underlying m-TSP, checking the capacity constraints with different levels of vehicle utilization. We discuss the constraint levels in Section 4.1.

#### 3.3.1. Feasibility Ratio

Feasibility Ratio (FsR) estimates the proportion of feasible solutions within the ground solution space (the solution space of the unconstraint base problem). Given a random walk of *n* points, the definition of FsR is as follows:

(16) $$FsR=\frac{n_{f}}{n}$$

where $n_{f}$ is the number of feasible points visited on the random walk of length *n*.

#### 3.3.2. Ratio of Feasible Boundary Crossings

Ratio of Feasible Boundary Crossings (RFBC) estimates the dispersion of feasible areas, quantifying the transitions between feasible and infeasible zones observed in a random walk. The principal hypothesis is that if infeasible areas separate many feasible ones, then there will be many transitions in a random walk. Given a sequence of *n* solutions generated by a random walk $\left(x_{1},x_{2},\ldots ,x_{n}\right)$, a binary sequence $\left(b_{1},b_{2},\ldots ,b_{n}\right)$ is constructed by assigning $b_{i}=1$ if the solution $x_{i}$ is feasible and $b_{i}=0$ otherwise. Thus, the measure is:

(17) $$\begin{array}{ccc} RFBC & = & \frac{\sum_{i=1}^{n-1}cross\left(i\right)}{n-1} \end{array}$$

(18) $$\begin{array}{ccc} cross(i) & = & \left\{\begin{array}{cc} 0 & \;\mathrm{if}\;b_{i}=b_{i+1}\\ 1 & \;\mathrm{if}\;b_{i}\neq b_{i+1} \end{array}\right. \end{array}$$

### 3.4. Fitness Landscape Analysis in Vehicle Routing Problems

The literature associated with FLA in VRP is relatively scarce compared to that of TSP ([19,20,21,22]). Kubiak [23] presents specific statistical FLA measures to analyze CVRP. Also, he argues that, based on his analysis of test instances, the local optima are close to each other, explaining why intensification strategies are successfully used in metaheuristics for CVRP. From a classification perspective, Czech [24] uses Simulated Annealing (SA) to explore the solution space to extract information from the fitness landscape to create a ranking of difficulty on the instances for Vehicle Routing Problem with Time Windows (VRPTW). Subsequently, from a comparative point of view, Runka & Ventresca [25,26] contrast mutation and crossover operators in CVRP and VRPTW. Van Stein [27] proposes an interesting but only exploratory analysis using Expanded Barrier Trees to relate the algorithm execution time (as a difficulty measure) of solving small (number of vehicles ≤6) instances with the vehicle capacity and the number of available vehicles. Similarly, Tian [28] proposes a study of inversion, relocate and exchange operators, although his results are limited to statistical analysis. Recently, Kovacs et al. [29] show that 2-opt dominates the global crossover operations in VRP. The experiments confirm the superiority of the 2-opt and Lin-Kernighan methods. Additionally, Agardi et al. [30] perform a type of FLA for the Multi-Echelon Vehicle Routing Problem. The article presents an information analysis approach and a detailed analysis of 2-opt, order crossover, cycle crossover and partially matched crossover based on distances. Based on the results, the 2-opt operator proved effective. On the other hand, Karkkainen and Rasku [31] propose a novel feature analysis and knowledge discovery process for Capacitated Vehicle Routing problems (CVRP) based on different features. Finally, we recommend Malan’s [32,33] periodic actualizations for updates on the advances in FLA.

### 3.5. Skewness of Information Content (SIC)

Although Vassilev [14] proposes to complement the classical statistical FLA measures with the perspective of information analysis, the FLA measures he proposes have not been used much. The main reason is the difficulty of comparing curves using a single scalar measure that gives a reduced (compact) representation of the curve, leaving aside the richness of its behavior. The only case where it has been done with much success is in statistical analysis with Correlation Length, which is a well-known scalar measure based on the behavior of the autocorrelation curve. In contrast, there is no positive evidence associated with measures describing the curves related to information analysis.

In the context of VRP, Pitzer [4] mentioned that information FLA measures could give definitive clues about particular problem instances, under certain circumstances. However, it seems almost impossible to create a direct interpretation by looking at the raw values (FLA measures at ε=0). Hence, we propose a new scalar measure that describe the curve of Information Content H(ε), that seem to represent better the shape of the entire curve (and not only a specific point as MIC). In particular, the new FLA measure allows to observe significant differences in the curves’ behavior, even when they have similar values for ε=0.

Section 3.2.3 presents the Information Content curve H(ε), in which the objects observed in the sequence change due to a modification of ε. Note that the signs computed with the function $\Psi_{f_{t}}\left(i,\varepsilon \right)$ to obtain the sequence s(ε) are determined by the relation between the difference of consecutive fitness values and the magnitude of ε. Consequently, to understand a little more about the fitness landscape’s structure, we can modify the epsilon value, which generates a change in the probability of occurrence of the different objects. Note that increasing the value of epsilon increases the number of neutral steps in the fitness landscape, as described in Section 3.2.3.

We propose to use a measure of skewness to compare the curves generated by different instances of a problem. Skewness of Information Content (SIC) considers that H(ε) is a sequence $\left\{H{\left(\varepsilon \right)}_{0}^{\varepsilon^{*}}\right\}$ for integer values of ε from 0 to $「\varepsilon^{*}」$, with the mean $\bar{H}$, where $\varepsilon^{*}$ is Stability. We describe the shape of the curve in terms of the second ($m_{2}$) and third ($m_{3}$) moments around the mean $\bar{H}$, using the Fisher-Pearson coefficient of skewness [34]:

(19) $$SIC=\frac{m_{3}}{m_{2}^{3/2}}\;\mathrm{with}\;m_{i}=\frac{1}{\varepsilon^{*}+1}\sum_{\varepsilon =0}^{\varepsilon^{*}}{\left(H\left(\varepsilon \right)-\bar{H}\left(\varepsilon \right)\right)}^{i}$$

**Figure 4:**
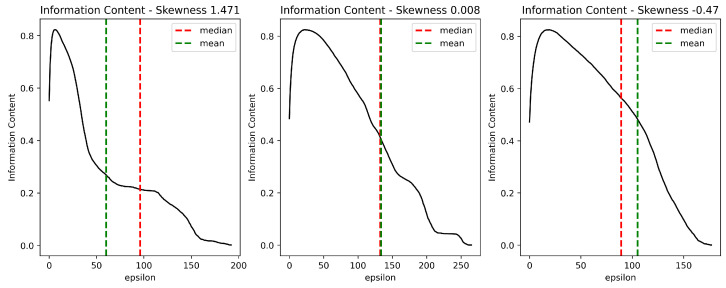
SIC values for different VRP instances. Note: although the values for ε=0 are similar for different instances, the curves’ shapes are significantly different.

Figure 4 shows that SIC gives an idea about the shape of the Information Content curve. In this context, SIC complements H(0) and MIC by characterizing the curve’s behavior throughout its domain. Note that the curve H(ε) is naturally right-skewed since MIC is generally reached at small epsilon values. Consequently, a negative SIC value indicates that rugged objects prevail over smooth objects with neutral steps in a broad segment of the H(ε) domain. In contrast, a positive SIC value indicates that both the variety and the number of rugged objects decrease rapidly.

## 4. Methods

Our research explores two main aspects of FLA in VRP: (1) to analyze the impact of the solution representation, local operator, and additional constraints on the properties of the underlying fitness landscapes and (2) to explain the difficulty for local search algorithms in terms of the fitness landscape’s properties. This section describes the main methodological aspects of the investigation.

### 4.1. Instances

Whereas real-life vehicle routing problems very often deal with instances where the total capacity of the fleet is much larger than the total demand, the instances studied in the literature associated with CVRP usually fix the number of vehicles close to $K=\frac{\sum_{i=1}^{n}d_{i}}{Q}$, where $\sum_{i=1}^{n}d_{i}$ is the total demand and *Q* is the vehicle capacity. This setting implies a very high fleet capacity utilization.

To explore the role of constraints, we propose new experimental instances based on 129 *base instances* taken from the literature ([35,36,37]), keeping the number of vehicles but varying the vehicle capacity to achieve different levels of fleet capacity utilization. First, we calculate the capacity $Q_{\max}$ for which the fleet achieves full use of its capacity. $Q_{\max}$ is computed as $\frac{\sum_{i=0}^{n}d_{i}}{K}$, and although $Q_{\max}$ is commonly infeasible, it is used as a reference point. From there, the capacity $Q_{\max}$ is relaxed to obtain $Q_{p}$ that corresponds to using p% of the fleet capacity. Note that $Q_{0}$ is equivalent to studying m-TSP with *K* vehicles, and we extend the analysis to study $Q_{20}$, $Q_{40}$, $Q_{60}$, and $Q_{80}$. In this way, we obtain the total of 645 *experimental instances*.

For example, consider an instance of CVRP with an original capacity Q=100, K=4 vehicles and the sum of all demands equal 300. The parameters associated with total demand, capacity and the number of vehicles generate the percentage of fleet capacity utilization of 75%. On the other hand, if Q=75, the capacity utilization is total. Thus, $Q_{\max}=75$ , $Q_{80}=93.75$, $Q_{60}=125$, $Q_{40}=187.5$, $Q_{20}=375$ and $Q_{0}\approx \infty$.

### 4.2. Representations and Fitness Landscape Exploration

In the context of FLA, any change (of solution space, neighborhood, or fitness function) in a fitness landscape’s structure affects an algorithm’s ability to search that space.

Search algorithms for VRP can use many alternative solution encodings. A Delimiters representation corresponds directly to a set of *k* individual routes. Notice that, like in VRP formulations based on three indexes, the routes/vehicles are distinguishable: the sub-permutation of customers between the first two occurrences of the depot correspond to the first vehicle, the customers between the second and the third occurrence correspond to the second vehicle, etc. In addition, notice a natural bijection between the solution space obtained using the Delimiters representation for m-TSP and the set of feasible solutions of our m-TSP MILP formulation. The same holds for the subspace of feasible solutions for CVRP and the feasible solutions of our CVRP MILP formulation.

In contrast, Giant-Tour representation corresponds to a TSP solution that needs to be partitioned into *k* individual routes to get a solution for VRP with *k* vehicles. The transformation is performed with a Split algorithm [38]: the permutation of all customers present in the Giant-Tour is split into a sequence of *k* sub-permutations, each consisting of customers that are consecutive on the Giant-Tour and maintaining the relative order of visits. If there are some ways of splitting the Giant-Tour into feasible routes (always true for m-TSP, but for CVRP only true if the demands of the customers can be assigned to the vehicles, respecting the vehicle capacity—thus only if there exists a solution to the corresponding Bin Packing problem), the Split algorithm chooses the one with the lowest cost. Otherwise, the Giant-Tour is infeasible. Notice that working with this representation implies that some of the feasible solutions for the MILP formulation of the corresponding VRP are not represented in the solution space. It can be easily seen by comparison with the Delimiters representation: given a fixed permutation of all customers, many different Delimiters representations are obtained by placing the k−1 delimiters, all copies of the depot except for the first and the last one, in all possible ways. On the other hand, working with the Giant-Tour representations, the Split algorithm will choose only one of them—one with the lowest cost (the highest fitness). In other words, many low fitness value VRP solutions are not present in the search space based on Giant-Tour.

**Figure 5:**
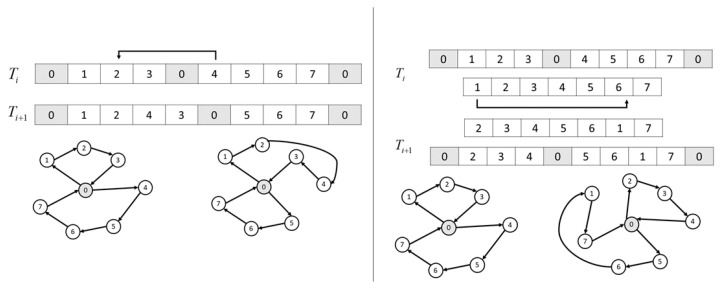
Representation of solutions in vehicle routing problems. (**Left**) Delimeters–(**Right**) Giant-Tour + Split Algorithm.

Figure 5 shows a relocation move in both representations. In Delimiters, the move is performed directly in the solution space. In contrast, the Giant-Tour representation involves decoding and encoding solutions. Note that the Split algorithm chooses the best feasible partitioning for the Giant-Tour.

The representations support application of the original operators developed for TSP (Giant-Tour) and their modifications for VRP (Delimiters). Furthermore, these types of representations are widely used in evolutionary and population-based methods.

Our analyses are based on sequences of solutions obtained from random walks in fitness landscapes. To create a sequence of solutions of the required length, we start with an empty sequence. First, we obtain the first solution by a random choice in the entire solution space and add it to the sequence. Then, we iteratively take the last solution from the sequence, choose one of its neighbors at random and add it to the sequence until the required length is achieved.

The procedure for obtaining the neighbor solution is quite different in the two representations. For Delimiters, given the current VRP solution, the local operator is applied at random to obtain a new VRP solution. If the new solution is feasible, then it is kept. Otherwise, we choose another solution at random. The choice is repeated until we find a feasible solution. We use this procedure for statistical and information analysis. In contrast, for the feasibility analysis, we accept all solutions. With the Giant-Tour representation, we apply the local operator in the TSP space, and the Split algorithm looks for the best feasible VRP solution using the new Giant-Tour.

To create the *Complete Dataset of Original FLA Measures*, we consider each experimental instance of CVRP (645 in total) with all combinations of representation and local operator, obtaining the total of 3870 fitness landscapes. For each fitness landscape, the corresponding FLA measures are extracted. We base statistical and information analysis on 30 random walks of 20,000 iterations. In contrast, the feasibility analysis considers a single large random walk of 100,000 iterations. Furthermore, we calculate SIC using a single random walk of 100,000 iterations.

### 4.3. Difficulty for Local Search

One of the main objectives in FLA is to find patterns or properties of a problem or instance that can be exploited to improve the performance of algorithms. In order to evaluate the performance of a particular algorithm, we need to have a close to optimum solution for each instance. To get them, we use an extension of Lin-Kernighan-Helsgaun [39] for m-TSP and CVRP. The benchmark results presented in the cited report indicate that the algorithm achieved the best known or new best solutions for 96.85% of m-TSP instances tested and 72.88% of CVRP instances. Note that the instances tested by Helsgaun [39] include those that are used in our research (up to 200 customers) and others with much larger numbers of customers (up to 30,000 customers for CVRP and up to 85,700 customers for mTSP).

Since the objective here is not to design a state of the art algorithm, but instead to explore the properties of fitness landscapes that influence the performance of local search based methods, we use one of the most straightforward and widely used algorithms for VRP: to evaluate the difficulty of instances for local search-based algorithms, we study the behavior of Simulated Annealing (SA).

SA converges towards an optimal solution, but it reaches this optimum only after an infinite number of search iterations. The approximation of the asymptotic behavior requires many iterations whose order of magnitude is equal to the state space’s cardinality. Nevertheless, the literature reports that good results can be obtained with a much smaller number of iterations.

Our simulations generate the initial solution from a permutation of the customers chosen uniformly at random (TSP solution space), by applying the Split algorithm. We propose an SA parametrization that uses an initial temperature T=1000 and the temperature varies across the iterations using a linear cooling schedule with a fixed cooling rate t=0.9 [5]. For each temperature, the algorithm performs $min(n^{2},10,000)$ iterations. To obtain a hardness measure, we calculate the Gap(%) between the average fitness value for solutions of SA ($f_{SA}$) and the best fitness value of LKH solutions ($f_{LKH}$) as:

(20) $$\mathrm{Gap}\%=100\times \frac{f_{SA}-f_{LKH}}{f_{LKH}}$$

$f_{SA}$ is calculated using ten runs per fitness landscape and $f_{LKH}$ is the best solution obtained in ten runs.

### 4.4. Finding Patterns with Machine Learning Techniques

Two objectives are essential for this proposal: (1) to explore differences between the fitness landscapes generated by alternative representations, local operators and levels of fleet utilization and (2) to find properties (or patterns of characteristics) of fitness landscapes that give clues about instances hard to solve with SA. In addition, we believe that lessons learned in our particular setting will help to design methodologies for other contexts.

Towards the first objective, we develop classification models based on Multinomial Logistic Regression (MLR) (The reader is referred to the textbook by Bishop [40] or the one by Murphy [41] for an introduction to machine learning.) to discover the local operator used to create a fitness landscape, given the underlying FLA measures. MLR is a commonly used classification model when more than two nominal classes are examined. This method is a generalization of the traditional Logistic Regression model to a non-binary case. For the second objective, we use the same methods to predict the performance of Simulated Annealing.

We perform the analyses on grouped datasets extracted from the Complete Dataset of Original FLA Measures. The description of the *grouped datasets* is as follows:

Task #1: Discover the local operator: Consider a partition based on representations. The Complete Dataset of Original FLA Measures is partitioned into the *Delimiters Dataset* and the *Giant-Tour Dataset*.

Task #2: Predict the performance of SA: Consider a partition based on local operators. Both, the Delimiters and Giant-Tour Datasets are subdivided into the *Delimiters (Giant-Tour, respectively) Relocate Dataset*, *Delimiters (Giant-Tour, respectively) Exchange Dataset* and *Delimiters (Giant-Tour, respectively) 2-Opt Dataset*.

To discover the local operator and predict the performance of simulated annealing, we decided to use grouped datasets based on the original variables to directly assess the importance of each metric proposed in the literature. A MLR model is built based on backward elimination as a feature selection technique.

In order to use classification models, we propose a discretization of the performance of SA in each grouped dataset. Consequently, we split the set of experimental instances into two equal-sized classes, based on the average Gap obtained by SA: Low (≤median) and High (>median). These categories let us capture performance behavior and explore underlying patterns associated with statistics, information and feasibility FLA measures.

**Figure 6:**
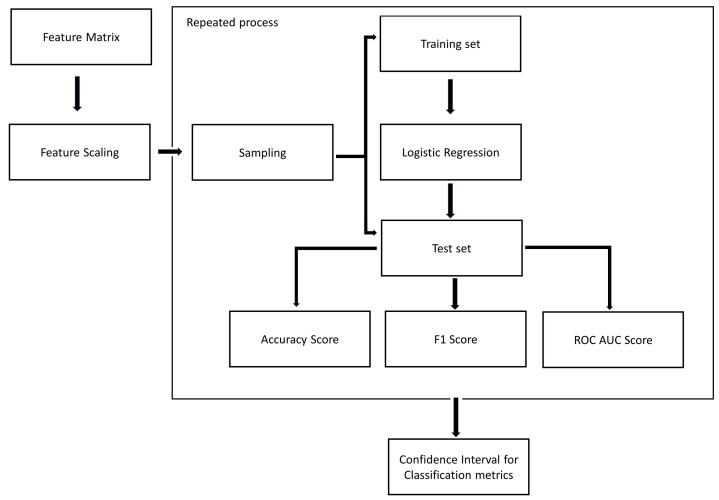
Steps of applying MLR.

As shown in Figure 6, we apply MLR over each grouped dataset and extract the performance evaluation metrics using a re-sampling technique. For each data mining task based on a grouped dataset, we use this method to create 100 *sample datasets* that contain only one occupancy rate for each base instance. In this way, we apply MLR with each sample and based on all the samples we obtain confidence intervals for the performance evaluation metrics in the classification problem.

Additionally, we perform a Principal Component Analysis (PCA) independently as a dimensionality reduction technique for each grouped dataset. For each dataset, PCA transforms the set of correlated variables into a smaller number of uncorrelated variables called *principal components* while retaining as much of the variation present in the datasets as possible. In this context, we select the principal components that capture at least 70% of the variability present in the data. Then, the projections onto the principal components are used as a *transformed* version of the grouped datasets. This procedure was performed previous to the MLR, to explore the relationships between the variables. However, these new components are not used in the classification models.

## 5. Results

To present the results (The reader is referred to explore the complete dataset [42]), we use the abbreviations IC, PIC and DBI to refer to the values of Information Content, Partial Information Content and Density-Basins Information at ε=0, respectively. Skewness of Information Content (SIC) is defined in Section 3.5 and describes the curve of Information Content. Additionally, Maximum Information Content (MIC), Correlation Length (CL), Feasible Solutions Ratio (FSR) and Ratio of Feasible Boundary Crossings (RFBC) are used.

### 5.1. Preliminary Results

#### 5.1.1. Constraints and Their Effect on FLA Measures

Table 1 shows the results of the Repeated Measure ANOVA [43] performed in the Complete Dataset of Original FLA Measures. Each cell represents the analysis for a combination of representation and operator (645 fitness landscapes), comparing the groups based on different fleet utilization levels (129 fitness landscapes, each). The analyses work with the following null ($H_{0}$) and alternative ($H_{A}$) hypotheses:

- $H_{0}$: The corresponding FLA measure at different utilization levels has the same population mean (i.e., $\mu_{mTSP}=\mu_{CVRP20}=\mu_{CVRP40}=\mu_{CVRP60}=\mu_{CVRP80}$).
- $H_{A}$: At least one population mean is different from the rest.

We do these analyses to know if different constraint levels lead to significantly different values of FLA measures. If the Fisher-test is significant (✓), it implies that at least two groups among those compared are significantly different (α≤0.05); otherwise (×) there is no evidence of a significant difference between the groups.

The results presented in Table 1 show that statistical and information FLA measures exhibit significant changes when a higher occupancy level yields a more constrained fitness landscape. Only for the Delimiters representation, in some cases, we obtain relatively invariant FLA measures. In the case of 2-Opt family, MIC does not change with a more constrained fitness landscape. For Exchange, the proposed measure SIC is invariable.

#### 5.1.2. Dimensionality and Its Effect on FLA Measures

One of the most relevant aspects in the study of VRP is the number of customers. Being a combinatorial problem, adding a new customer presents a substantial increase in the problem’s dimensionality.

As shown in Table 2, IC and MIC present decreasing trends when the number of nodes increases. In contrast, CL, PIC and DBI show increasing trends. These results are largely due to lower frequencies of neutral steps observed in sequences sampled from fitness landscapes based on instances with more customers. In general, one can observe in our samples that VRP lead to fitness landscapes composed mainly of rugged and smooth objects without neutral steps (at ε=0). The feasibility FLA measures, FSR and RFBC, also exhibit negative correlations. We found no evidence of a relationship between the proposed measure (SIC) and the number of nodes.

In the following subsections, we explore (1) the potential of using standard FLA measures and the one that we propose to differentiate between fitness landscapes obtained by using different representations and local operators and (2) the possibility to use information, statistical and feasibility FLA measures to predict the difficulty of searching the fitness landscape of a VRP instance with Simulated Annealing.

### 5.2. Exploratory Analysis of the Fitness Landscape

#### 5.2.1. Structure Differences between Local Operators by Representation

Some authors [14,44] claim that different operators lead to different fitness landscapes. Unfortunately, the capacity of FLA measures to describe these differences is not widely explored in the literature. For this reason, we propose to use PCA to explore the differences between fitness landscapes based on FLA measures. We conduct this analysis separately on Delimiters and Giant-Tour Datasets, transforming the datasets with PCA as mentioned in Section 4.4.

Table 3 presents the loading matrix with a Varimax rotation that represents the correlations between the original variables and the principal components.

For Delimiters Dataset, we obtain the following three principal components:

Delimiters Principal Component 1 (DPC-1) is strongly correlated with IC, PIC and SIC. A positive value in PC-1 indicates a high IC, a high SIC and a low PIC. A high value of IC, which implies a high frequency or diversity of rugged objects, combined with a low value of PIC, which indicates in the context of high IC a high number of neutral steps, indicates a relatively large amount of rugged objects with neutral steps. On the other hand, the positive relationship with SIC indicates that as ε increases, rugged objects without neutral steps rapidly change to include neutral steps. That is, the differences in fitness between neighbors are low. Conversely, a low value in DPC-1 indicates a lower diversity of rugged objects (low frequency of neutral steps, high number of peaks), increasing the PIC value and generating larger differences between fitness values at nearby solutions.

Delimiters Principal Component 2 (DPC-2) is positively related to CL and DBI, and negatively to MIC. A high value in DPC-2 indicates that, comparatively, an instance achieves a lower MIC value. Furthermore, the positive relationship with DBI confirms a more significant presence and diversity of non-rugged objects. In this context, the relationship with CL indicates that smoother Landscapes will have a higher value in DPC-2.

Delimiters Principal Component 3 (DPC-3) is positively related to RFBC and negatively correlated to FSR. This indicates that instances with a high value in DPC-3 have many transitions between feasible and infeasible solutions and a low proportion of feasible solutions.

In contrast, for Giant-Tour Dataset, we obtain the following four principal components:

Giant-Tour Principal Component 1 (GPC-1) has the same interpretation as the DPC-2 component in the Delimiters case. However, the percentage of explained variance of the data is much higher (32.8%). The result indicates that unlike Delimiters, mainly explained by the behavior of rugged objects, the variability of Giant-Tour Dataset is focused on smooth objects. For Giant-Tour, objects without neutral steps are even more likely than for Delimiters (at ε=0). Since the Giant-Tour representation works on a smaller search space, based on solutions of TSP, with the single tour divided into k tours in a unique way through the split algorithm, the probability of two neighboring solutions to have the same fitness value tends to be even lower than with Delimiters.

Giant-Tour Principal Component 2 (GPC-2) has a similar interpretation to the DPC-1 component of Delimiters representation. Nevertheless, it does not include SIC.

Giant-Tour Principal Component 3 (GPC-3) is positively related to SIC and negatively to RFBC. In this way, as mentioned in the description of GPC-2, the information provided when studying the Information Content variations when epsilon increases is independent from IC and PIC (at ε=0). GPC-3 summarizes the variability of the route’s length and fleet utilization level. In general, an instance that presents small differences in fitness values (high SIC) and fleet utilization level between neighbors, the latter translating into fewer transitions between feasible and infeasible zones (low RFBC), will have a high value in GPC-3. On the other hand, note that there is a low correlation of GPC-3 with the FLA measures directly related to the smoothness of the fitness landscape, which suggests that despite having small differences between neighboring solutions (with respect to both distance and occupancy), a fitness landscape could be intuited as rugged if using only traditional FLA measures. Finally, unlike for Delimiters, where RFBC appears only in conjunction with FSR (in DPC-3), for Giant-Tour RFBC provides some information independent of FSR, which indicates that among a group of instances that present similar values of FSR, the infeasible areas may be highly fragmented in some (high RFBC) and compact in others (low RFBC).

Giant-Tour Principal Component 4 (GPC-4) is related to FSR and RFBC, as is DPC-3 in the Delimiters case.

**Figure 7:**
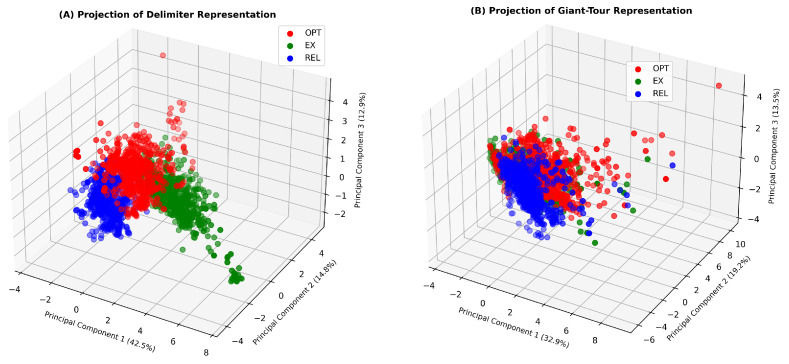
Projection of instances onto the principal components. (**A**) Projection for delimiters representation and (**B**) Projection for Giant-Tour representation.

The projection in Figure 7 shows strong differences between the local operators for both representations. For Delimiters, most of the variability is concentrated in the first component. Thus, Relocate presents low (negative) values in DPC-1, indicating a fitness landscape that presents a greater number of peaks than 2-Opt and Exchange, respectively. On the other hand, the projection of the observations on the principal components for Giant-Tour shows smaller differences compared to Delimiters (explained variance of components).

#### 5.2.2. Difficulty for Local Search by Local Operator and Representation

This analysis is performed separately on 6 grouped datasets: Delimiters (Giant-Tour, respectively) Relocate Dataset, Delimiters (Giant-Tour, respectively) Exchange Dataset and Delimiters (Giant-Tour, respectively) 2-Opt Dataset.

As mentioned in Section 4.4, we propose to partition the fitness landscapes in each grouped dataset into two equal-sized classes, based on the average Gap obtained by SA: Low (≤median) and High (>median). The classes are created from the performance of a particular operator and representation without differentiating between occupancy rates. For Delimiters, the 2-Opt Family is categorized as having a high Gap when it exceeds a Gap of 4.27%; In Relocate and Exchange, the categorizations are made taking Gaps of 6.11% and 20.47% as thresholds, respectively. Likewise, in Giant-Tour, the difficulty categories are established at the Gap of 3.72% for 2-Opt, 3.35% for Relocate and 8.93% for Exchange. The above gives an idea of the differences obtained using a specific local operator or representation.

We perform PCA on the grouped data sets proposed for the task #2.

**Figure 8:**
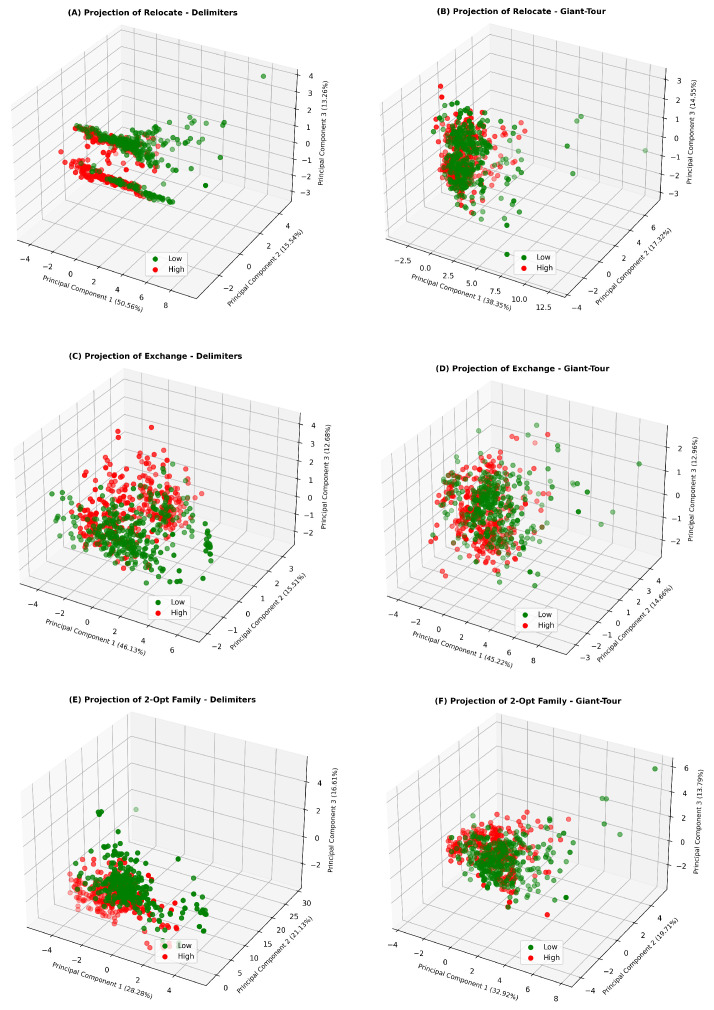
Projection of instances with high or low Gap onto the principal components—based on different configurations.

Figure 8 shows a difference between the two types of representations, since the three local operators studied present more separability in the representation of Delimiters. On the other hand, there is a greater difficulty in separating instances with a high or low Gap in the Giant-tour representation. Thus, the use of more sophisticated tools such as MLR could contribute to the identification of patterns that are not distinguishable using simple dimensionality reduction mechanisms.

### 5.3. Task #1 : Discover the Local Operator

To classify the operators, we use MLR. In this task, this model is used to discover the local operator that was used to obtain a given fitness landscape, based on FLA measures.

Using Delimiters and Giant-Tour Datasets and applying the re-sampling technique, we split each sample dataset (recall from Section 4.4, 100 in total) into training (90 instances equivalent to 70%) and test (39 instances equivalent to 30%) sets. The training set is used to fit the MLR and the test set is used to extract the classification metrics: Accuracy, F1 and ROC AUC scores. The results for the samples are used to build a confidence interval (95%) for the classification metrics.

As presented in the subsection on preliminary results, we expect that a machine learning algorithm could identify with high accuracy the local operators for Delimiters. This preliminar idea is confirmed with the performance evaluation metrics shown in Table 4. For both representations, MLR presents good performance in classifying the local operators. However, the performance of the classification algorithm is noticeably lower for Giant-Tour. This result was expected given the more complex structure of the neighborhoods.

Also, as shown in Table 5, all the variables are significant for the model based on Delimiters representation. In contrast, for the LR based on Giant-tour representation, only IC, SIC, CL and FSR are significant. Note that SIC is significant in both models. In this way, we conclude that a good description of the information content curve gives more information to identify the local operator that generates the fitness landscape.

### 5.4. Task #2 : Predict the Difficulty for Local Search

One of the main challenges in combinatorial optimization and algorithm design is to understand the characteristics of a problem or instance that causes difficulties in finding good solutions.

**Figure 9:**
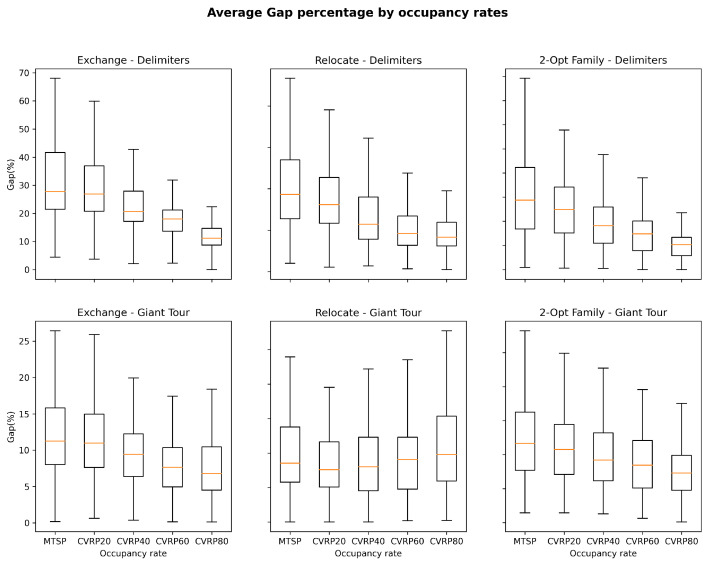
Average Gap over different fleet utilization levels.

In the case of CVRP, we observe significant differences in Simulated Annealing’s performance when changing occupancy rates, local operators and representations. As shown in Figure 9, a more constrained fitness landscape provides more favorable search conditions in Delimiters, regardless of the local operator being studied. The results could be explained by the decrease in the size of the search space. A similar behavior, although less prominent, can be observed for Giant-Tour, with exception of the Relocate operator. On the other hand, the Delimiters representation leads to higher values of Gap than Giant-Tour, which could be explained by the fact that the sizes of search spaces are significantly larger when using Delimiters.

As shown in Table 6, MLR achieves good results for most representations and local operators. However, the quality of models is lower for Giant-Tour, which could indicate that the FLA measures that we use to analyze the performance of local search may not be sufficient for the analysis of fitness landscapes generated by more complex exploration mechanisms.

Additionally, we show in Table 7 that CL and FSR are significant for all the models. On the contrary, the Ratio of Feasible Boundary Crossings is only present in the models concerning a fitness landscape based on the Relocate operator. Also, the proposed measure SIC is part of a few models. The result indicates that in some cases, a description of the H(ε) curve is important to describe the performance of SA.

## 6. Conclusions

The VRP instances commonly used in the literature correspond to very high occupancy rates [20,35,45]. In contrast, in industry practice, VRPs often operate with moderate occupancy rates. Therefore, conclusions based on historical analyses (with high occupancy rates) might not apply to industry cases. We note that this limitation could strongly influence certain FLA measures, narrowing them down to specific patterns. Consequently, we focus our research on the study of FLA measures for instances with different occupancy rates. In this context, based on the results of repeated measures ANOVA, we show variations in the behavior of the FLA measures at different occupancy rates.

In an exploratory analysis, we extract principal components from the datasets. In this way, we establish that many of the FLA measures proposed by Vassilev do not generate additional information when analyzed at epsilon = 0. On the other hand, most of the variability of the Delimiters Dataset is based on FLA measures associated with the fitness landscape’s ruggedness, which is contrasted with the Giant-Tour results, in which most of the data variability set is based on FLA measures of smoothness.

Additionally, we analyze the ability of the FLA measures to differentiate the local operators with which the fitness landscape is built (for a fixed representation). The results establish that for the representation of Delimiters, MLR can differentiate between the local operators, based on the FLA measures, with high accuracy. However, the quality of models decreases considerably when the Giant-Tour representation is analyzed, indicating that the FLA measures we consider might not be sufficient for the study of representations and operators that yield more complex neighborhood structures.

We explore different encodings (Delimiters and Giant-Tour) and local operators (Exchange, Relocate and 2-Opt Family) and verify that they lead to different characteristics in the fitness landscape. We find that FLA measures based on statistics, information and feasibility analyses can identify some patterns in the fitness landscape generated by each configuration (representation-operator) and relate them to the difficulty of obtaining good solutions with a local-search-based algorithm (in our case, the Simulated Annealing).

Finally, an essential contribution of this paper is the unification of different techniques of Fitness Landscape Analysis in the context of Vehicle Routing Problems and the proposal of the SIC measure as a new way to describe the Information Content curve. Our results add new information about fitness landscapes’ structure that can be useful for developing new methods and understanding their behavior.

## Figures and Tables

**Table 1:** Results for Repeated Measures ANOVA for FLA measures per occupancy rates.

	Relocate		2-Opt Family		Exchange	
**Measures**	**DEL**	**G-T**	**DEL**	**G-T**	**DEL**	**G-T**
IC	✓	✓	✓	✓	✓	✓
DBI	✓	✓	✓	✓	✓	✓
PIC	✓	✓	✓	✓	✓	✓
MIC	✓	✓	×	✓	✓	✓
CL	✓	✓	✓	✓	✓	✓
SIC	✓	✓	✓	✓	×	✓
FSR	✓	✓	✓	✓	✓	✓
RFBC	✓	✓	✓	✓	✓	✓

**Table 2:** The Pearson Correlation Coefficient (ρ) between the FLA measures and the number of nodes. Each cell corresponds to a FLA measure for some representation and local operator. We calculate ρ using an aggregation based on the average behavior in the occupancy rates. * denotes the coefficients that are not significant at α=0.05.

	Relocate		2-Opt Family		Exchange	
**Measures**	**DEL**	**G-T**	**DEL**	**G-T**	**DEL**	**G-T**
IC	−0.704	−0.346	−0.462	−0.543	−0.775	−0.623
DBI	0.814	0.649	−0.030 *	0.615	0.498	0.758
PIC	0.659	0.279	0.349	0.452	0.717	0.543
MIC	−0.747	−0.679	−0.090	−0.607	−0.613	−0.694
CL	0.936	0.966	0.938	0.945	0.932	0.988
SIC	0.088 *	−0.012 *	0.098 *	0.073 *	−0.014 *	0.148 *
FSR	−0.709	−0.756	−0.671	−0.766	−0.585	−0.753
RFBC	−0.681	−0.822	−0.638	−0.839	−0.478	−0.827

**Table 3:** Loading matrix with Varimax Rotation. Correlation between principal components and original variables. High values are highlighted in bold.

Dataset	Delimiters			Giant-Tour			
**Measures**	**DPC1**	**DPC2**	**DPC3**	**GPC1**	**GPC2**	**GPC3**	**GPC4**
IC	**0.904**	−0.232	−0.008	0.138	**0.979**	−0.019	−0.044
PIC	**−0.879**	0.222	0.051	−0.058	**−0.979**	−0.009	0.032
SIC	**0.824**	−0.027	0.036	0.109	0.066	**0.833**	0.235
MIC	0.414	**−0.756**	−0.067	**0.833**	0.063	0.069	0.038
DB	−0.465	**0.702**	−0.047	**−0.782**	−0.251	−0.002	−0.029
CL	0.007	**0.838**	−0.035	**−0.817**	−0.071	0.049	0.055
FSR	−0.047	−0.020	**−0.814**	0.044	0.124	−0.139	**−0.880**
RFBC	−0.131	−0.065	**0.629**	0.249	0.204	**−0.589**	**0.485**
% Exp.	42.5%	14.8%	12.9%	32.8%	19.2%	13.5%	12.7%
% Cum.			70.2%				78.2%

**Table 4:** Classification summary for MLR to identify local operators based on FLA measures.

Metric	Delimiters Representation	Giant-Tour Representation
Accuracy	0.979 ± 0.006	0.831 ± 0.013
F1 Score	0.979 ± 0.006	0.828 ± 0.013
Roc Auc Score	0.995 ± 0.002	0.936 ± 0.007

**Table 5:** Significance of the variables for MLR to discover the local operator. Statistically significant variables (α=0.05) are represented with (✓).

Representation	IC	PIC	SIC	MIC	DB	CL	FSR	RFBC
Delimiters	✓	✓	✓	✓	✓	✓	✓	✓
Giant Tour	✓	×	✓	×	×	✓	✓	×

**Table 6:** Classification summary for MLR to identify the difficulty of local search.

Represent.	Operator	Accuracy	F1 Score	ROC AUC
Delimiters	Relocate	0.81 ± 0.01	0.81 ± 0.01	0.87 ± 0.01
Delimiters	Exchange	0.79 ± 0.01	0.78 ± 0.01	0.86 ± 0.01
Delimiters	2-Opt Fam.	0.82 ± 0.01	0.80 ± 0.01	0.89 ± 0.01
Giant-Tour	Relocate	0.79 ± 0.01	0.78 ± 0.01	0.86 ± 0.01
Giant-Tour	Exchange	0.77 ± 0.01	0.76 ± 0.01	0.83 ± 0.01
Giant-Tour	2-Opt Fam.	0.71 ± 0.01	0.71 ± 0.01	0.78 ± 0.01

**Table 7:** Significance of the variables for MLR to identify difficulty of instances. Statistically significant variables (α=0.05) are represented with (✓).

Repr.	Operator	IC	PIC	SIC	MIC	DB	CL	FSR	RFBC
DEL	Relocate	✓	✓	×	✓	×	✓	✓	✓
DEL	Exchange	✓	✓	×	×	✓	✓	✓	×
DEL	2-Opt Fam.	✓	✓	✓	✓	✓	✓	✓	×
G-T	Relocate	×	×	✓	✓	×	✓	✓	✓
G-T	Exchange	✓	✓	×	×	✓	✓	✓	×
G-T	2-Opt Fam.	×	✓	✓	×	×	✓	✓	×

## Data Availability

The complete dataset is available [42].
